# Supplementary material for: A Global Screen for Assembly State Changes of the Mitotic Proteome by SEC-SWATH-MS
Source: Cell Syst. 2020 Feb 26;10(2):133–155.e6. doi: 10.1016/j.cels.2020.01.001 (PMC7042714; doi:10.1016/j.cels.2020.01.001)

Q5BJH7 | YIF1B_HUMAN | YIF1B PP4519 UNQ3073/PRO9905
Monomer MW [kDa]: 34.435 Monomer expected elution fraction: 49

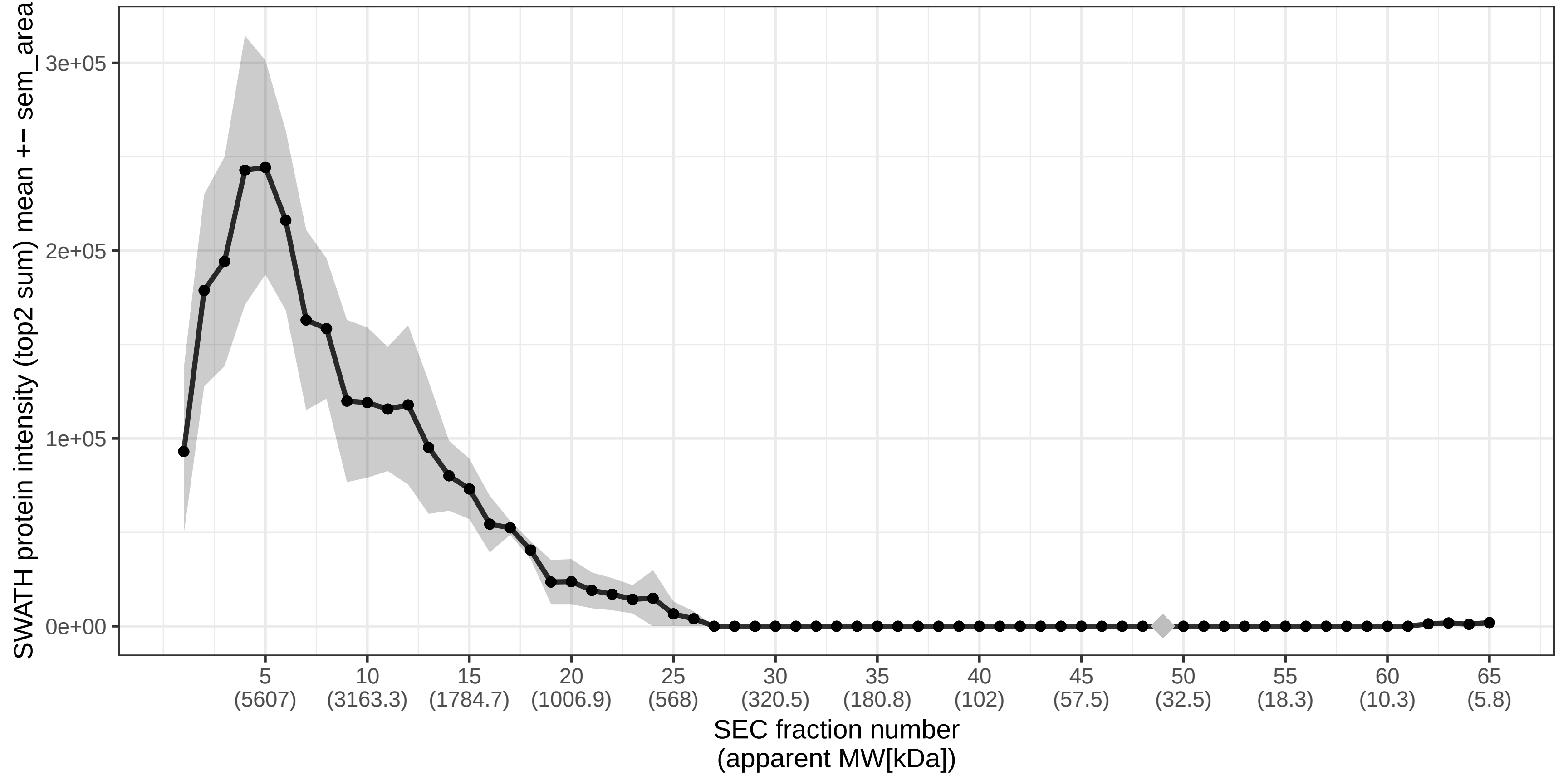

Supplement: Data S1. SEC-SWATH-MS Protein Chromatograms, Related to Figure 1 [file mmc6.zip › SECchrom_Q5BJH7_YIF1B_HUMAN_YIF1B_PP4519_UNQ3073_PRO9905.pdf]
